# Analyzing subcomponents of affective dysregulation in borderline personality disorder in comparison to other clinical groups using multiple e-diary datasets

**DOI:** 10.1186/s40479-016-0039-z

**Published:** 2016-07-06

**Authors:** P. S. Santangelo, M. F. Limberger, C. Stiglmayr, M. Houben, J. Coosemans, G. Verleysen, P. Kuppens, F. Tuerlinckx, W. Vanpaemel, U. W. Ebner-Priemer

**Affiliations:** Karlsruhe Institute of Technology, Karlsruhe, Germany; Consortium for Scientific Psychotherapy Berlin (AWP-Berlin), Berlin, Germany; KU Leuven – University of Leuven, Leuven, Belgium; Central Institute of Mental Health, Mannheim, Germany

**Keywords:** Borderline personality disorder, Affective dysregulation, Specificity, E-diary, Electronic diary, Ambulatory assessment

## Abstract

**Background:**

Affective dysregulation is widely regarded as being the core problem in patients with borderline personality disorder (BPD). Moreover, BPD is the disorder mainly associated with affective dysregulation. However, the empirical confirmation of the specificity of affective dysregulation for BPD is still pending. We used a validated approach from basic affective science that allows for simultaneously analyzing three interdependent components of affective dysregulation that are disturbed in patients with BPD: homebase, variability, and attractor strength (return to baseline).

**Methods:**

We applied two types of multilevel models on two e-diary datasets to investigate group differences regarding three subcomponents between BPD patients (*n =* 43; *n =* 51) and patients with posttraumatic stress disorder (PTSD; *n =* 28) and those with bulimia nervosa (BN; *n =* 20) as clinical control groups in dataset 1, and patients with panic disorder (PD; *n =* 26) and those with major depression (MD; *n =* 25) as clinical control groups in dataset 2. In addition, healthy controls (*n =* 28; *n =* 40) were included in the analyses. In both studies, e-diaries were used to repeatedly collect data about affective experiences during participants’ daily lives. In study 1 a high-frequency sampling strategy with assessments in 15 min-intervals over 24 h was applied, whereas the assessments occurred every waking hour over 48 h in study 2. The local ethics committees approved both studies, and all participants provided written informed consent.

**Results:**

In contradiction to our hypotheses, BPD patients did not consistently show altered affective dysregulation compared to the clinical patient groups. The only differences in affective dynamics in BPD patients emerged with regard to one of three subcomponents, affective homebase. However, these results were not even consistent. Conversely, comparing the patients to healthy controls revealed a pattern of more negative affective homebases, higher levels of affective variability, and (partially) reduced returns to baseline in the patient groups.

**Conclusions:**

Our results indicate that affective dysregulation constitutes a transdiagnostic mechanism that manifests in similar ways in several different mental disorders. We point out promising prospects that might help to elucidate the common and distinctive mechanisms that underlie several different disorders and that should be addressed in future studies.

## Background

Affective dysregulation is widely regarded as being the core problem in patients with Borderline Personality Disorder (BPD) and the driving force behind the severe clinical manifestations of BPD symptoms. This is supported by a magnitude of empirical findings relating affective dysregulation to other BPD symptoms and behaviors [1–3]. The importance of affective dysregulation is furthermore delineated in the DSM-5 [4] and the ICD-10 [5], since several diagnostic criteria for BPD, such as, e.g., affective instability, intense anger, and chronic feelings of emptiness directly reflect aspects of dynamic affective difficulties. In one of the most highly regarded theories of BPD, the biosocial theory [6], the affective dysregulation emerges from transactions between biological vulnerabilities and specific environmental influences. According to this model the affective dysregulation in BPD manifests in: (a) a high sensitivity to emotional stimuli (especially negative ones) resulting in a lower threshold for responding to those; (b) intense responses to emotional stimuli, i.e., emotional responses with greater amplitudes; and in (c) a longer duration of emotional responses and thus a slow return to baseline after responding to emotional stimuli. In a nutshell, affective dysregulation is of central importance in this disorder and has had major impacts on theory, research, and treatment. However, most recently Santangelo et al. [7] used an electronic diary approach to clarify the specificity of affective instability in BPD, and largely failed. BPD patients showed comparable affective instability to patients with posttraumatic stress disorder (PTSD) and patients with bulimia nervosa (BN), which raised the question to consider subcomponents of affective dynamics.

To further clarify the unsatisfying findings regarding the specificity of affective instability and to delineate differences in emotional processes between patients with BPD and clinical controls, Ebner-Priemer et al. [8] proposed the use of the DynAffect model [9], i.e., a model from basic affective science. The DynAffect model synthesizes different lines of basic research on emotion dynamics into one coherent model with the objective of identifying the major processes that underlie individual differences in the temporal dynamics of affective experiences [9]. It assumes three components which characterize individual differences in affect dynamics. These postulated components can be mapped to the three subcomponents of affective dysregulation defined in the biosocial theory of BPD [6] as shown above: (a) affective homebase, i.e., an individual baseline attractor state around which affect fluctuates; (b) level of affective variability, i.e., the fluctuations around the homebase that result from responses to internal or external processes or events; and (c) attractor strength, i.e., regulatory or homeostatic processes that pull deviating affective fluctuations back toward the homebase and thus enables emotional recovery. Dysregulated affect can become expressed in several ways in the three DynAffect components. First, negative deviations from a normative affective homebase that is mildly positive and aroused [10] can be indicative of affect dysregulation. Second, high levels of affect variability are related to lower psychological well-being [11], and are considered to be, at least to some extent, the result of disrupted emotion regulation [12]. Third, high attractor strength reflects successful affect regulation with affective experience being strongly pulled back to the homebase, whereas low attractor strength indicates affect that keeps lingering, suggestive of failing affect regulation efforts [13].

Ebner-Priemer et al. [8] empirically validated the appropriateness of the DynAffect model in examining affective dysregulation in BPD by statistically modeling data from three e-diary studies containing data of patients with BPD and healthy subjects. This validation showed strong support for more negative affective homebases and heightened affective variabilities as well as partial support of a slower return to baseline in BPD patients compared to healthy controls, both with regard to valence as well as distress. Thus, Ebner-Priemer et al. [8] presented a theoretical model that captures the most fundamental affective dynamical processes that are supposed to underlie BPD and showed the usefulness of this model by applying it to multiple empirical datasets. However, the question regarding the specificity of the three subcomponents of affective dysregulation was not addressed in this study, since the datasets did not include any clinical control groups.

To address the still open question whether affective dysregulation is specific for patients with BPD or whether affective dysregulation rather constitutes a transdiagnostic feature of several mental disorders, we simultaneously analyzed the three subcomponents of affective dysregulation (homebase, variability, and attractor strength). We analyzed two independent datasets to investigate whether BPD patients show a specific pattern of affective dysregulation as proposed by the biosocial theory of BPD [6]. We compared BPD patients (*n =* 43 and *n =* 51) with clinical control groups consisting of patients with PTSD (*n =* 28) and with BN (*n =* 20) in dataset 1, and patients with panic disorder (PD; *n =* 26) and with major depression (MD; *n =* 25) in dataset 2. In both studies, e-diaries were used to repeatedly collect data about affective experiences (valence and distress) during participants’ daily lives. In study 1 we applied a high-frequency sampling strategy with assessments of momentary valence and distress in 15 min-intervals over 24 h during waking time. In the second study, we utilized a sampling strategy with repeated assessments of current distress every waking hour over 48 h. We applied two types of multilevel models to simultaneously analyze the subcomponents of affective dysregulation [8] as proposed by the biosocial theory [6]. Due to the predictions derived from the biosocial theory, we hypothesized (a) a more negative affective homebase in the BPD group compared to the clinical control groups; (b) BPD patients to exhibit heightened affective variability in comparison to the clinical control groups; and (c) that the patients with BPD would exhibit reduced attractor strength (i.e., a higher autocorrelation reflecting a slower return to baseline) compared to the clinical control groups. In addition, we included healthy control participants in the analyses to check robustness of the results.

## Methods

### Participants: dataset 1

A total sample of 119 female participants between 18 and 48 years of age was investigated: 43 patients with BPD, 28 patients with PTSD, 20 patients with BN and 28 healthy controls. Data were collected at the Central Institute of Mental Health Mannheim and the Psychosomatic Clinic St. Franziska-Stift Bad Kreuznach in Germany. Outpatients and inpatients were recruited from their outpatient clinics or wards or via advertisements in local newspapers and on the internet. The healthy controls were selected randomly from the national resident register of the City of Mannheim or recruited via advertisement. The participants’ sample characteristics are summarized in Table 1. For further details about the dataset, please consult the publication of Santangelo et al. [7] describing the specific aspects of it. With regard to this dataset, statistical analyses of other characteristics have been reported. In details, the specificity of global instability [7] as well as first analyses regarding subcomponents of affective dysregulation comparing BPD patients to healthy controls [8]. However, the main research question of the current paper, namely if subcomponents of affective dysregulation show specificity for BPD patients compared to clinical controls, have not been analyzed or reported before.

**Table 1:** Demographic Characteristics of dataset 1 and dataset 2

	Dataset 1 (*N* = 119)				Dataset 2 (*N* = 142)			
	BPD (*n* = 43)	PTSD (*n* = 28)	BN (*n* = 20)	HC (*n =* 28)	BPD (*n* = 51)	PD (*n* = 26)	MD (*n* = 25)	HC (*n* = 40)
Age in years								
Mean (Sd)	26.7 (7.1)	35.25 (7.5)	23.70 (6.0)	28.82 (7.5)	27.1 (6.7)	33.1 (8.4)	34.1 (7.8)	27.8 (7.4)
Sex								
% female	100	100	100	100	100	100	100	100
Total number of e-diary data entries								
Mean (Sd)	57.91 (7.7)	58.50 (8.7)	56.70 (7.7)	56.68 (7.1)	26.3 (3.4)	24.5 (4.0)	23.5 (3.3)	26.0 (3.3)
	BPD (*n* = 43)	PTSD (*n* = 28)	BN (*n* = 20)		BPD (*n* = 51)	PD (*n* = 26)	MD (*n* = 25)	
Psychotropic medication								
*n* (%)	16 (37 %)	17 (60 %)	5 (25 %)		22 (43 %)	10 (39 %)	24 (96 %)	
Hospitalization								
Outpatients *n* (%)	26 (60 %)	8 (29 %)	9 (45 %)		26 (51 %)	21 (81 %)	13 (52 %)	
Inpatients *n* (%)	17 (40 %)	20 (71 %)	11 (55 %)		25 (49 %)	5 (19 %)	12 (48 %)	
Current Axis I diagnoses *n* (%)								
Major depression	9 (21 %)	15 (54 %)	10 (50 %)		17 (33 %)	4 (15 %)	25 (100 %)	
Anxiety disorders	27 (63 %)	19 (68 %)	10 (50 %)		35 (69 %)	26 (100 %)	12 (48 %)	
Generalized anxiety disorder	6 (14 %)	1 (4 %)	1 (5 %)		8 (16 %)	5 (19 %)	4 (16 %)	
Panic disorder	14 (33 %)	10 (36 %)	2 (10 %)		13 (26 %)	26 (100 %)	2 (8 %)	
Agora phobia	3 (7 %)	0 (0 %)	2 (10 %)		4 (8 %)	23 (89 %)	1 (4 %)	
Other phobias	20 (47 %)	15 (54 %)	9 (45 %)		16 (31 %)	5 (19 %)	6 (24 %)	
Posttraumatic stress disorder	22 (51 %)	28(100 %)	3 (15 %)		7 (14 %)	0 (0 %)	6 (24 %)	
Obsessive-compulsive disorder	5 (12 %)	0 (0 %)	1 (5 %)		6 (12 %)	0 (0 %)	2 (8 %)	
Eating disorders	14 (33 %)	20 (71 %)	20 (100 %)		18 (35 %)	0 (0 %)	1 (4 %)	
Current Axis II disorders *n* (%)								
Cluster A	7 (16 %)	3 (11 %)	1 (5 %)					
Cluster B (besides BPD diagnosis)	3 (7 %)	0 (0 %)	1 (5 %)					
Cluster C	26 (61 %)	8 (29 %)	4 (20 %)					

*BPD* Borderline Personality Disorder, *PTSD* Posttraumatic Stress Disorder, *BN* Bulimia Nervosa, *PD* Panic Disorder, *MD* Major Depression, *HC* Healthy Controls

### Participants: dataset 2

Sample 2 consists of 142 female participants between 17 and 50 years of age: 51 BPD patients, 26 PD patients, 25 MD patients and 40 healthy controls. Data were collected at the Freiburg University Medical School and at the Free University of Berlin, both located in Germany. Outpatients and inpatients were recruited from their outpatient clinics and private practices or wards, respectively. The healthy controls were randomly selected from the national resident register of the City of Freiburg. The sample characteristics are shown in Table 1. For a more detailed description of the dataset, please consult the publication of Stiglmayr et al. [14]. There are no previous publications on this data set with regard to affective instability or affective dysregulation as in dataset 1.

### Diagnostic procedure: dataset 1 and 2

All patients met the DSM-IV criteria for their specific disorder. In both samples, Axis I disorders were assessed using the German version of the Structured Clinical Interview for DSM-IV Axis I Disorders (SCID-I; [15]), and Axis II disorders were assessed using the German versions of the International Personality Disorder Examination (IPDE; [16]) in dataset 1 and the BPD section of the Structured Clinical Interview for DSM-IV Axis II Disorders (SCID-II; [17]) in dataset 2, respectively. In the healthy control group, the absence of any current or past Axis I or Axis II disorder diagnoses was confirmed by the SCID-I and SCID-II. Trained postgraduate psychologists administered all diagnostic instruments. In the patient groups, a history of schizophrenia or bipolar disorder or current substance abuse constituted exclusion criteria. Furthermore, patients of the clinical control groups who met the criteria for BPD were excluded in both studies. All other comorbidities were allowed in the patient groups. The exclusion criteria for the healthy controls included any current or past Axis I or Axis II disorder diagnoses, self-reported current psychotherapy, or the current use of psychotropic medications. As Table 1 shows, patients with BPD had very high rates of comorbid Axis I disorders, particularly eating disorder, anxiety disorders, and depressive disorders in both datasets. Whereas patients with PTSD and those with BN had similar high rates of comorbid eating, anxiety, and depressive disorders in dataset 1, the patients with PD and those with MD had lower rates of comorbidities, at least with regard to eating disorders in dataset 2.

### Data collection procedure: datasets 1

All participants provided written informed consent prior to participation in the study, which has been approved by the local ethics committee. Participants were carefully instructed and trained regarding the use of the palmtop computer (Tungsten E, Palm Inc., U.S.A.). To function as e-diaries the palmtop computers were programmed with the DialogPad software (Gerhard Mutz, Cologne University, Germany). Subsequent to the training session, participants carried the e-diary for 24 h. The e-diary emitted a prompting signal every 15 min (±1 min) during the waking time. Questions regarding the participants’ current emotions followed each prompt. Participants were asked “Do you feel any of the following emotions right now?” followed by the list of happy, anxious, angry, shame, disgust, sad, guilt, interest, envy/jealousy, emotion but cannot name it, and no emotion. After selecting a current emotional state, participants rated the intensity of this emotion on an 11-point Likert scale ranging from 1 to 11. In case that “emotion but cannot name it” was chosen, an additional question was added concerning the pleasantness of the current emotion (pleasant or unpleasant) followed by the intensity rating. In addition, participants rated their current intensity of distress on an 11-point Likert scale from 0 to 10. After the 24-h assessment period participants returned the device and the data were downloaded from the e-diaries.

### Data collection procedure: datasets 2

The local ethics committees approved the study, and all participants provided written informed consent before participating. Participants attended an orientation session to get familiar with the use of the palmtop computer (Psion 3a, Psion PLC, United Kingdom). The palmtop computers were programmed with the MONITOR software [18] to emit a prompting signal in hourly intervals (±5 min). Participants carried the e-diary over a 48-h period and were prompted every hour during waking time to provide information regarding their current subjective distress experience on a single 10-point Likert scale ranging from 0 to 9. After the 48-h assessment period participants returned the devices and the data were downloaded from the e-diaries.

### Compliance: dataset 1 and 2

Compliance in both datasets was very high (94 % and 92 %, respectively). Participants in dataset 1 provided on average 57.55 (Sd = 7.77; Median = 57) self-reports, whereas participants in dataset 2 provided on average 25.37 (Sd = 3.58; Median = 26) momentary assessments (Table 1; for a more detailed description of compliance see Santangelo et al. [7] for dataset 1 and Stiglmayr et al. [14] for dataset 2).

## Data preprocessing and statistical analyses

### Data preprocessing

To aggregate the separate assessments of emotion and intensity into a single valence index in dataset 1, the intensities of negative emotions were multiplied by −1, and the intensities of the positive emotions retained positive values [7]. This method resulted in valence scores with a range of −11 to +11. Ratings of “no emotion” were given valence scores of zero. Thus, the two dependent variables used in the statistical analyses were (a) valence (with possible values ranging from −11 to +11) and (b) distress (with possible values ranging from 0 to 10). No data preprocessing was necessary in dataset 2. Possible values of distress as the dependent variable ranged from 0 to 9.

For the subcomponents analysis of distress, data from one BPD patient and one PTSD patient and eight healthy controls were excluded from the analyses due to lack of variability or linear trend in their ratings in dataset 1. The final sample for this analysis consisted of 42 BPD patients, 27 patients with PTSD, 20 patients with BN and 20 healthy controls. No participants were excluded in the analysis of valence in this dataset. In dataset 2, two patients with MD and seven healthy controls were excluded from the analyses due to lack of variability in their distress ratings. Thus, the final sample consisted of 51 BPD patients, 26 patients with PD, 23 patients with MD and 33 healthy controls.

### Statistical analyses

We used multilevel modeling to analyze the three subcomponents of affective dysregulation: (a) affective homebase (i.e., one’s affective baseline state), (b) affective variability (i.e., the total sum of the fluctuations around the affective homebase in response to internal or external events), and (c) attractor strength (i.e., the regulatory processes that pull affect back to its homebase or return to baseline). These statistical approaches were used to simultaneously model the three different parameters and to investigate how these parameters differed as functions of group (BPD vs. PTSD vs. BN and BPD vs. PD vs. MD, respectively).

The main analyses were performed using HLM [19]. To test our hypotheses, a total of three models with the BPD group as the reference group were tested (i.e., one model with valence and one with distress as outcome variables in dataset 1 and one model with distress as the outcome variable in dataset 2). The multilevel regression analyses models were defined as follows (with the BPD patients as the reference group):

Level 1 equation:

$$ \mathrm{distres}{\mathrm{s}}_{\mathrm{ti}}={\uppi}_{0\mathrm{i}}+{\uppi}_{1\mathrm{i}}\ast \kern0.28em \mathrm{distres}{{\mathrm{s}}_{\left(t-1\right)}}_{\mathrm{i}}+{\mathrm{e}}_{\mathrm{ti}} $$

Level 2 equation:

$$ \begin{array}{l}{\uppi}_{0\mathrm{i}}={\beta}_{00}+{\beta}_{01}\kern0.28em \ast \left(\mathrm{P}\mathrm{T}\mathrm{S}{\mathrm{D}}_{\mathrm{i}}\right)+{\beta}_{02}\ast \left(\mathrm{B}{\mathrm{N}}_{\mathrm{i}}\right)+{\mathrm{r}}_{0\mathrm{i}}\\ {}{\uppi}_{1\mathrm{i}}={\beta}_{10}+{\beta}_{11}\ast \left(\mathrm{P}\mathrm{T}\mathrm{S}{\mathrm{D}}_{\mathrm{i}}\right)+{\beta}_{12}\ast \left(\mathrm{B}{\mathrm{N}}_{\mathrm{i}}\right)+{\mathrm{r}}_{1\mathrm{i}}\\ {}\mathrm{V}\mathrm{a}\mathrm{r}\left(\mathrm{R}\right)={\sigma}^2\mathrm{and} \log \left({\sigma}^2\right)={\upalpha}_0+{\upalpha}_1\left(\mathrm{P}\mathrm{T}\mathrm{S}{\mathrm{D}}_{\mathrm{i}}\right)+{\upalpha}_2\left(\mathrm{B}{\mathrm{N}}_{\mathrm{i}}\right)\end{array} $$

where distress_ti_ corresponds to the distress rating for person *i* at time *t*. At level 1, distress_ti_ is predicted by a random intercept and a time-lagged version of itself (i.e., distress_(t-1)i_). This variable has been centered around the person mean (i.e., within-person centered) and previous-day observations were set as missing to exclude day-to-day carry-over effects. The random slope of this lagged variable, π_1i_, is the autoregressive effect of distress_(t-1)i_ on distress_ti_. At level 2, the random intercept and slope are both predicted by an intercept and two dummy variables coding for the clinical control groups. The equation shown is the multilevel model comparing the BPD group (as the reference group it is not represented by a dummy variable) to the PTSD group and the BN group. β_00_ corresponds to the mean distress, i.e., the distress homebase, in the BPD group (when the PTSD and BN dummies are equal to zero). Similarly, β_10_ corresponds to the mean autoregressive slope, i.e., the return to baseline, in the BPD group (when the two dummy coded group variables are zero). Simultaneously, the within-person variance is modeled as a function of the two dummy variables. For the examination of valence, distress_ti_ was replaced by valence_ti_ (dataset 1). For the analyses in dataset 2 the dummy variables coding for the clinical groups were replaced (i.e., PTSD_i_ and BN_i_ were replaced by PD_i_ and MD_i_). Additionally, we estimated three models with the same outcome variables, this time examining general differences between healthy controls and clinical groups. More specifically, we estimated a multilevel model with the healthy controls as the reference group and a dummy coding for all patients taken together (i.e., the healthy controls vs. BPD + PTSD + BN in dataset 1, and the healthy controls vs. BPD + PD + MD in dataset 2, respectively).

**Fig. 1:**
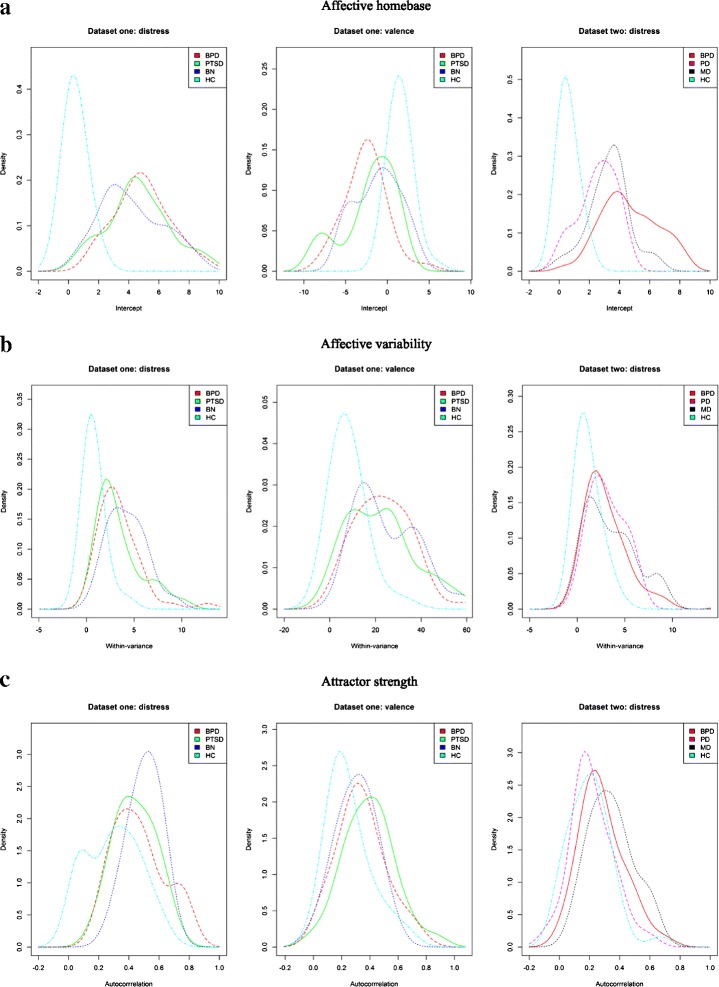
Density plots based on the models applied in the Bayesian framework. The plots depict the distributions of the posterior mean estimations of the three coefficients corresponding to the components of affective dysregulation with regard to distress and valence. Distributions of the respective coefficient estimates (coefficient *intercept *corresponding to the subcomponent affective homebase in **a**, coefficient *within-variance *corresponding to the subcomponent within-person variability in **b**, and  coefficient *autocorrelation *corresponding to the subcomponent attractor strength in **c**) are shown for each investigated group separately for dataset 1 (patients with borderline personality disorder [BPD], those with posttraumatic stress disorder [PTSD], those with bulimia nervosa [BN] and healthy controls [HC]) and dataset 2 (patients with borderline personality disorder [BPD], those with panic disorder [PD], those with major depression [MD] and healthy controls [HC])

Last, in order to enable the identification of consistent patterns of results and to allow for robust conclusions we repeated all analyses with slightly different models and in a different statistical framework. These models are extensions of the models used before, in that not only the intercept and the autoregressive slope but also the within-person variance was estimated as a random effect. In other words, we allowed for individual differences in within-person variances, while the models described above assume that the within-person variance is homogenous within diagnostic groups. The statistical inference for these models was done using Bayesian statistics instead of the frequentist statistical approach that is adopted in the models described above. Bayesian statistics requires the specification of priors. For each person the model has a person-specific intercept, slope and within-person variance (the latter is log-transformed). The triplet of person-specific parameters is assumed to come from a trivariate normal population distribution. This trivariate distribution has a population mean for each parameter that may differ across the clinical groups. We used dummy coding schemes to incorporate these group differences. Both for the reference group mean as for the deviations of the other groups, we assumed as priors relative vague normal distributions with mean being zero and variance being 1000 (for the autocorrelation, the normal prior distributions were truncated at −1 and 1). The variance-covariance matrix of the trivariate normal distribution was the same for all groups and its prior was an inverse-Wishart with an identity matrix as scale matrix and four degrees of freedom. The Bayesian analyses were implemented in JAGS [20]. We used four chains with a burn-in period of 5000 iterations. The subsequent 2500 iterations from each chain were used for inference (no thinning). Convergence of the chains was checked for the different parameters visually (i.e., by looking at the trace plots and the autocorrelation graphs), and numerically (i.e., by calculating the shrink factor) [21, 22]. The trace plots were stationary time series, and they overlapped for different chains, while the autocorrelation plots did not indicate any strong autocorrelation. For all parameters, the shrink factor was very close to 1 (and always below 1.1). Taken together, these diagnostics suggest good convergence. Moreover, based on these additional models applied in the Bayesian framework we also constructed density plots (see Fig. 1a, b, and c). For each specific parameter (i.e., homebase, variability, and attractor strength), we first estimated the posterior mean per person and then we made a density plot for each group based on these posterior means, showing the (posterior) distributions of the parameters of interest for each investigated group separately.

## Results

### Subcomponent affective homebase

Results from the multilevel regression models are shown in Table 2. These indicated that the distress level of the homebase of the BPD patients was comparable to that of the PTSD and BN patients in dataset 1 (HOMEBASE, π_0_, Table 2). BPD patients and those with PTSD also had homebases characterized by similar levels of unpleasantness, whereas the BPD patients had a homebase that was marginally significantly more negative compared to the BN patients. Using multilevel models applied in the Bayesian framework, no differences were found between BPD and the clinical groups for both the valence and the distress dimension of the homebase (i.e., the marginally significant difference between the BPD and the BN patients with regard to valence was not robust). In dataset 2, the BPD patients had a homebase with significantly higher distress levels compared to the PD and MD patients, which was confirmed by the models applied in the Bayesian framework.

**Table 2:** Estimates from multilevel models for the three subcomponents of affective dysregulation with patients with borderline personality disorder as the reference group

	Dataset 1											Dataset 2				
	Distress					Valence						Distress				
	Coeff.	SE	Test statistic	df	*p*	Coeff.	SE	Test statistic	df	*p*		Coeff.	SE	Test statistic	df	*p*
For HOMEBASE, π_0_											For HOMEBASE, π_0_					
Intercept, β_00_	4.91	0.284	17.28	86	<.001	–2.59	0.410	–6.33	88	<.001	Intercept, β_00_	4.66	0.275	16.92	97	<.001
PTSD_i_, β_01_	–0.23	0.497	–0.46	86	.65	0.50	0.743	0.68	88	.50	PD_i_, β_01_	–2.06	0.373	–5.54	97	**<.001**
BN_i_, β_02_	–0.71	0.552	–1.29	86	.20	1.43	0.759	1.88	88	.06	MD_i_, β_02_	–1.30	0.415	–3.15	97	**<.001**
For VARIANCE											For VARIANCE					
Intercept, α_0_	1.22	0.032	38.32		<.001	3.14	0.031	99.65		<.001	Intercept, α_0_	1.05	0.044	23.90		<.001
PTSD_i_, α_1_	–0.03	0.050	–0.70		.48	–0.06	0.049	–1.31		.19	PD_i_, α_1_	0.02	0.078	0.24		.81
BN_i_, α_2_	0.19	0.056	3.41		<.001	0.08	0.056	1.44		.15	MD_i_, α_2_	0.09	0.084	1.12		.26
For AUTOREGRESSIVE slope, π_1_											For AUTOREGRESSIVE slope, π_1_					
Intercept, β_10_	0.46	0.036	12.51	86	<.001	0.33	0.034	9.48	88	<.001	Intercept, β_10_	0.26	0.038	6.87	97	<.001
PTSD_i_, β_11_	–0.02	0.052	–0.35	86	.73	0.04	0.056	0.63	88	.53	PD_i_, β_11_	–0.04	0.064	–0.58	97	.56
BN_i_, β_12_	0.04	0.048	0.86	86	.39	–0.04	0.057	–0.71	88	.48	MD_i_, β_12_	0.08	0.076	1.09	97	.28

*PTSD* Posttraumatic Stress Disorder, *BN* Bulimia Nervosa, *PD* Panic Disorder, *MD* Major Depression

Bolded values = significant difference supported by the result of the multilevel model with Bayesian inference

Moreover, patients showed a homebase with significantly higher levels of distress compared to healthy controls in dataset 1 and 2 (HOMEBASE, π_0_, Table 3). Furthermore, the healthy controls tended to have a pleasant valence homebase, whereas the clinical groups, patients with BPD, those with PTSD and those with BN were, on average, characterized by a significantly more unpleasant or negative valence homebase in dataset 1. All results were in line with the conclusions from the multilevel models with Bayesian inference.

**Table 3:** Estimates from multilevel models for the three subcomponents of affective dysregulation with healthy controls as the reference group

	Dataset 1											Dataset 2				
	Distress					Valence						Distress				
	Coeff.	SE	Test statistic	df	*p*	Coeff.	SE	Test statistic	df	*p*		Coeff.	SE	Test statistic	df	*p*
For HOMEBASE, π_0_											For HOMEBASE, π_0_					
Intercept, β_00_	0.47	0.114	4.10	107	<.001	1.53	0.233	6.58	117	<.001	Intercept, β_00_	0.58	0.094	6.24	131	<.001
BPD + PTSD + BN_i_, β_01_	4.21	0.242	17.43	107	**<.001**	–3.65	0.389	–9.34	117	**<.001**	BPD + MD + PD_i_, β_01_	3.24	0.215	15.10	131	**<.001**
For VARIANCE											For VARIANCE					
Intercept, α_0_	–0.24	0.045	–5.26		<.001	2.11	0.039	54.49		<.001	Intercept, α_0_	0.05	0.054	0.97		.33
BPD + PTSD + BN_i_, α_1_	1.49	0.050	29.69		**<.001**	1.03	0.044	23.23		**<.001**	BPD + MD + PD_i_, α_1_	1.03	0.063	16.29		**<.001**
For AUTOREGRESSIVE slope, π_1_											For AUTOREGRESSIVE slope, π_1_					
Intercept, β_10_	0.28	0.059	4.71	107	<.001	0.25	0.037	6.74	117	<.001	Intercept, β_10_	0.18	0.044	3.97	131	<.001
BPD + PTSD + BN_i_, β_11_	0.18	0.063	2.89	107	**<.01**	0.07	0.044	1.69	117	.09	BPD + MD + PD_i_, β_11_	0.10	0.053	1.86	131	.07

*BPD* Borderline Personality Disorder, *PTSD* Posttraumatic Stress Disorder, *BN* Bulimia Nervosa, *PD* Panic Disorder, *MD* Major Depression

Bolded values = significant difference supported by the result of the multilevel model with Bayesian inference

The distributions of the affective homebase parameters for all groups in dataset 1 and 2 are depicted in the density plots shown in Fig. 1a. Descriptively, the impressive differences between the healthy controls and the clinical groups hit the eye in all three figures of Fig. 1a. For healthy controls the plots are more located to the left for distress in both datasets (indicating lower distress levels), and more to the right for valence (reflecting a more positive homebase). Moreover, the estimates are more homogenous within the healthy control sample, as the plots are less wide. In contrast, only minor differences among the clinical groups seem to emerge, with the only exception of the distribution of the homebase parameters for distress in dataset 2. The plot for the BPD patients is located more to the right and is broader, reflecting higher levels of distress and more heterogeneity within the BPD sample.

### Subcomponent within-person variability

Results showed that the within-person variability (VARIANCE, Table 2) of distress in the BPD patients was comparable to that of the PTSD patients (dataset 1) and the PD and MD patients (dataset 2). The BN patients in dataset 1 had a significantly elevated variability of distress compared to the BPD patients, albeit this significant difference was not robust (since the results of the models with Bayesian inference indicated no difference of within-person variability of distress between BN and BPD patients). With regard to the variability of valence no significant differences between BPD patients and PTSD or BN patients was evident, which was supported by the results of the multilevel models with Bayesian inference.

The results for the variability of distress and valence revealed significant differences between healthy controls and all patient groups. Patients with BPD and those with PTSD and with BN showed significantly higher within-person variability of distress as well as valence compared to healthy controls in dataset 1 (VARIANCE, Table 3). Heightened variability in distress compared to the healthy subjects was also found in patients (those with BPD, those with PD and those with MD) in dataset 2. Again, these results were in line with the conclusions from the multilevel models with Bayesian inference.

On a descriptive basis, clear differences emerged between the healthy controls and the clinical groups in the density plots in Fig. 1b. The density plots for the healthy controls are again located more to the left, reflecting lower average within-person variance for this group. However, the differences between the clinical groups seem negligible.

### Subcomponent attractor strength (return to baseline)

No differences emerged comparing the attractor strength between the clinical groups, neither between the BPD group and the PTSD and BN patients (dataset 1) nor between the BPD group and the PD and MD patients (dataset 2) and neither regarding attractor strength in distress nor valence (AUTOREGRESSIVE slope, π_1_, Table 2).

The results regarding attractor strength of distress indicate a significantly lower autoregressive slope (i.e., higher attractor strength and thus faster return to baseline) in the healthy controls compared to BPD, PTSD and BN patients (AUTOREGRESSIVE slope, π_1_, Table 3) in dataset 1. The result from the model with Bayesian inference is in line with this finding. In dataset 2, marginally significantly higher attractor strength in distress was found in the healthy controls when compared to the patient groups. However, this difference was not found in the models applied in the Bayesian framework. For valence, we only found marginally significantly higher attractor strength for healthy controls in comparison to the patient groups. Again, this difference was not found in the models with Bayesian inference.

The density plots for the attractor strength component are not as clear as those for the other two components, since the distributions of the healthy controls do not seem to clearly stand out (see Fig. 1c). The only exception is the plot for the attractor strength parameter for distress in dataset 1, since the distribution for healthy controls is slightly more located to the left (indicating higher attractor strength). With regard to differences between the clinical groups, there are only slight differences between the distributions of the attractor strength estimates.

## Discussion

To further clarify the specificity of affective dysregulation in BPD, we used a recently proposed model of affective dynamics (the DynAffect model) simultaneously modeling three central subcomponents of affective dysregulation (homebase, variability, and attractor strength). Contrary to our expectations, BPD patients did not consistently show a specific pattern of affective dysregulation compared to other clinical groups (i.e., patients with PTSD, with BN, with PD and with MD). Therefore, our results indicate that affective dysregulation is, apart from very few exceptions, not very specific for BPD, as the clinical groups tended to show similar results. In dataset 1 we found no robust differences (i.e., consistent differences between the groups in both types of multilevel analyses) neither regarding the distress level of the homebases nor the valence dimension of the homebases of the BPD patients and the PTSD and BN patients. Furthermore, we did not find consistent results regarding elevated affective variability nor slower return to baseline in the BPD patients compared to either patient group, i.e., PTSD and BN patients (dataset 1) or PD and MD patients (dataset 2). The only robust exception (i.e., with consistent findings in both types of multilevel models) where patients with BPD showed altered affective dynamics compared to the clinical controls was: the BPD patients had a homebase with significantly higher levels of distress compared to the PD and the MD patients (dataset 2). Thus, the only differences emerged with regard to one of three subcomponents. Furthermore, the results regarding the homebase component were not perfectly consistent, since: (a) differences regarding the distress dimension of the homebase were only found in dataset 2 (i.e., the PD and the MD patients), but not in dataset 1 (i.e., the PTSD and BN patients); and (b) no differences regarding the valence dimension of the homebase emerged. Taken together, our results do not show specificity of affective dysregulation regarding several components of affective dysregulation (i.e., homebase, variability, and attractor strength) for patients with BPD. Instead, our results can be interpreted as further empirical evidence for affective dysregulation manifesting in similar ways in several different disorders that are characterized by affective disturbances.

Of course, BPD is the disorder mainly associated with affective dysregulation and there is even a BPD journal with emotion dysregulation it its title. However, there are multiple theoretical conceptualizations which associate a variety of mental disorders with affective dysregulation, i.e., some kind of burdensome affective experience, deficient affect regulation, or dysfunctional affect regulation behavior [23–27]. This is in line with the idea that affect regulation is an essential component to mental health [9, 28] and an important risk and maintaining factor in various mental disorders [29], and that affect regulation strategies are included as treatment modules across numerous disorders, e.g., eating disorders [30, 31], depressive disorders [32], and PTSD [33]. Taken together, these results might be interpreted as an indication that affective dysregulation rather constitutes a transdiagnostic feature that emerges in several mental disorders. Moreover, the differences between the patient groups and the healthy controls regarding all three subcomponents of affective dysregulation are greatly consistent, both for distress and valence. In a similar vein, prior studies investigating the specificity of affective instability for BPD revealed mixed findings regarding heightened instability in BPD compared to clinical controls. While several diary studies found heightened affective instability in BPD compared to patients with depressive disorders [34–36], no differences were found between BPD patients and patients with PTSD [7], those with BN or with anorexia nervosa [7, 37], patients with premenstrual dysphoric syndrome [34], or other personality disorders [38]. Therefore, global instability indices were not able to clearly differentiate the clinical groups and thus instability did not show sufficient specificity. Due to the unexpected nature of these findings analyzing subcomponents of the dynamic processes in order to delineate existing differences in emotional processes between patients with BPD and clinical controls has been proposed recently [7, 8]. However, this could not resolve inconsistencies as we have shown in the present paper. Thus, even though we used state of the art assessment (e-diaries) and analytic methods (multilevel modeling) as well as two comprehensive datasets (*N =* 119 and *N =* 142) we did not find clear group differences regarding the subcomponents of affective dysregulation between BPD patients and patients with PTSD, those with BN, those with PD and those with MD as clinical control groups.

As we can exclude with reasonable certainty that subcomponent analyses reveal specificity of affective dysregulation for BPD there are, on a methodological level, two more main topics that should be considered to differentiate affective processes between disorders [7, 26]: (a) events and triggers of emotional episodes are rarely assessed, but are very likely to differ between disorders (a notable exception in BPD is [39]). E.g., tempting food might trigger affective processes in patients with BN, but not in patients with PTSD, whereas for traumatic memories the opposite pattern might be expected. Moreover, context plays a central role in emotion regulation [40, 41]. Therefore, contextual factors should be systematically incorporated into the study of emotion dysregulation in future studies.; (b) the appraisal of affective processes might be worthy of examination, since affective changes that are accompanied by changes in self-esteem (a further diagnostic criterion for BPD) might be experienced as more threatening [7]. Thus, the association between affective instability and self-esteem instability in patients with BPD might differ (and therefore be specific for BPD) from those with other psychiatric disorders. This association between affective dysregulation and self-esteem instability in patients with BPD and those with other psychiatric disorders should be investigated in future studies.

On top of that, undifferentiated affect or emotional granularity has been discussed as being an essential component of the affect regulation process [42, 43]. However, its potential to show specificity of altered affect in BPD patients seems rather limited, since a recent study showed that the experience of undifferentiated affect probably constitutes a transdiagnostic mechanism and might be likely relevant to a range of disorders [44].

### Limitations and methodological particularities

Some limitations of our study should be mentioned. We used electronic diaries to investigate affective dysregulation in participants’ everyday lives. This comes along with the disadvantage that the control of confounding variables is limited. Even though laboratory studies offer the possibility of testing hypotheses under the most rigorous control, they nonetheless do so under artificial, laboratory conditions, which may account for differences between the laboratory and real life [45–47]. Investigating affective dysregulation in everyday life has the crucial advantage that it renders experimental symptom induction unnecessary: it is studied in the context where it naturally occurs, in patients’ everyday life [48]. Studies that have examined affective dysregulation in BPD in the laboratory have produced inconsistent findings, which might be explained by the affect induction methods used in these studies [8]. A further advantage of e-diary assessments is that retrospective single assessments such as questionnaires or interviews are not suited to investigate dynamic processes, such as affective dysregulation [26, 48–51]. By utilizing e-diary methods one can repeatedly assess the variable of interest in real time and therefore actually track the ebb and flow of affective states.

When investigating affective dynamics using e-diaries, it is of primary importance that the sampling rate matches the temporal dynamics of the underlying target process [26, 48, 52]. Both a sampling rate that is too infrequent (which might miss the dynamics of interest) as well as a sampling rate that is too frequent (which might overburden participants without increasing insights since the information is irrelevant) is problematic [53]. Even though guidelines regarding the sampling frequency are rare, there is a general consensus that the sampling interval must fit the temporal dynamics of the target processes [52, 54]. In our two datasets the time-based designs differed. Assessments occurred every 15 min in datasets 1, and every hour in dataset 2. Since the conclusions were largely similar across both datasets, the assessment methods do not seem to have substantially influenced the results. Moreover, we are confident that both sample designs were appropriate to assess the affective dynamics, since it has been empirically shown that a sampling interval of less than 1 h captures a specific process, whereas the data yielded by low frequency sampling rates (i.e., 2 h intervals and longer) cannot be distinguished from random data [52].

Even though both the assessment method and the sample frequencies are appropriate to investigate affective dynamics, the cross-sectional design of study 1 and 2 renders it impossible to evaluate the importance of the affective dysregulation in the long term. We determined the three subcomponents of affective dysregulation only during 24 h and 48 h, respectively. Thus, we got only a snapshot of affective dysregulation. This is adequate to analyze group differences regarding affective dysregulation; however, it is inappropriate to investigate potential associations between affective dysregulation and long term variables, such as level of functioning and symptom severity, psychopathology, or treatment outcome. Therefore, longitudinal studies allowing for the investigation of the predictive value of affective dysregulation are clearly needed.

A further limitation is the rather small sample sizes of the clinical control groups in both datasets. Even though both datasets were extensive (*N =* 119 and *N =* 142, respectively), this was mainly due to large group sizes of the BPD patients and the healthy controls. With regard to the clinical groups, the group sizes of 28 patients with PTSD, 20 patients with BN and 26 PD patients and 25 MD patients are low and larger sample sizes are needed to replicate findings. However, prior studies analyzed group differences based on as small group sizes as 15 patients with BPD and four patients with anorexia nervosa [37], or 16 patients with BPD, 10 patients with MD and 15 patients with premenstrual dysphoric syndrome [34]. Moreover, the patient groups in both datasets differed in their hospitalization rates. However, no differences in symptom severity between hospitalized and non-hospitalized patients emerged (see Santangelo et al. [7] for dataset 1 and Stiglmayr et al. [14] for dataset 2). Furthermore, because only female participants were included in both datasets, the generalizability of the findings is constrained and the results may not be valid for male BPD patients. However, the use of a pure female sample also reduced heterogeneity, which may be useful given the literature on sex differences on affect [55]. In study 1 and 2 BPD diagnoses were made using different diagnostic instruments, i.e., IPDE [16] in study 1 and SCID-II [17] in study 2. However, both diagnostic instruments are well-validated with very good psychometric properties and good interrater reliability [16, 17]. Moreover, the two datasets were analyzed separately and independently, thus, diagnoses and group comparisons are valid within each study. Patients, especially BPD patients, in both datasets were diagnosed with a variety of comorbid disorders. Given the finding that comorbidity might alter affective dysregulation [56] no statement can be made on whether our findings are independent of any comorbidity. However, in BPD comorbidity is the rule rather than the exception [57] and therefore, only BPD patients with comorbid disorders are seen as representative for the BPD population [58].

## Conclusions

In summary, using sophisticated behavioral science research methodology and validated analytic techniques we were unable to reveal a specific pattern of affective dysregulation in patients with BPD compared with clinical controls (i.e., patients with PTSD, those with BN, those with PD or those with MD). Even though affective dysregulation is widely regarded as being the core problem in patients with BPD and BPD being the disorder mainly associated with affective dysregulation, our results are in accordance with clinical observations and reports that most psychological disorders show some kind of affective dysregulation. Taken together, these findings suggest that affective dysregulation might be an important clinical characteristic of several disorders, in the sense of a transdiagnostic symptom or risk factor. Nonetheless, the possibility of evaluating the importance of a construct such as affective instability in a cross-sectional study is clearly restricted. Longitudinal studies allowing for the investigation of the predictive value of affective dysregulation might reveal group differences. Thus, even though affective instability does not show specificity for BPD it might be the case that it can be used to predict symptom severity, psychopathology, or treatment outcome in BPD whereas it might be of minor relevance in other patient groups. Addressing the association between affective dysregulation and self-esteem instability or events and triggers of emotional episodes in patients with BPD and those with other psychiatric disorders constitute auspicious approaches for future studies in order to reveal specific patterns of symptom expression in BPD.

## Abbreviations

BPD = Borderline Personality Disorder; PTSD = Posttraumatic Stress Disorder; BN = Bulimia Nervosa; PD = Panic Disorder; MD = Major Depression; HC = Healthy Controls; SCID-I = Structured Clinical Interview for DSM-IV Axis I Disorders; SCID-II = Structured Clinical Interview for DSM-IV Axis II Disorders; IPDE = International Personality Disorder Examination
